# Diversity of core promoter elements comprising human bidirectional promoters

**DOI:** 10.1186/1471-2164-9-S2-S3

**Published:** 2008-09-16

**Authors:** Mary Qu Yang, Laura L Elnitski

**Affiliations:** 1 National Human Genome Research Institute, National Institutes Health, Rockville, MD 20852, USA

## Abstract

**Background:**

Bidirectional promoters lie between adjacent genes, which are transcribed from opposite strands of DNA. The functional mechanisms underlying the activation of bidirectional promoters are currently uncharacterised. To define the core promoter elements of bidirectional promoters in human, we mapped motifs for TATA, INR, BRE, DPE, INR, as well as CpG-islands.

**Results:**

We found a consistently high correspondence between C+G content, CpG-island presence and an average expression level increasing the median level for all genes  in bidirectional promoters. These CpG-rich promoters showed discrete initiation patterns rather than broad regions of transcription initiation, as are typically seen for CpG-island promoters. CpG-islands encompass both TSSs within bidirectional promoters, providing an explanation for the symmetrical co-expression patterns of many of these genes. In contrast, TATA motifs appear to be asymmetrically positioned at one TSS or the other.

**Conclusion:**

Our findings demonstrate that bidirectional promoters utilize a variety of core promoter elements to initiate transcription. CpG-islands dominate the regulatory landscape of this group of promoters.

## Background

The complexities of promoter regions are slowly being revealed with help from a series of groundbreaking studies on vast collections of promoter sequences [1]. Proximal promoter regions (~500 bp upstream and 100 bp downstream of the TSS) typically contain the features necessary for basal levels of gene expression. Within the proximal promoter region, core promoter elements (CPEs) such as TATA, CCAAT, the initiator element (INR), TFIIB recognition element, downstream promoter element (DPE), represent distinct functional entities, along with CpG-islands, responsible for basal promoter activity. Computational studies of large collections of promoters classify them by these components, either individually or in combination. Thus far discrete functional mechanisms have not been fully elucidated for each class of promoter. However patterns of transcription initiation have been defined for CpG-islands, which are typically broad stretches of DNA with numerous start sites, and for TATA box motifs, which have single well-defined start sites [1].

A relatively new category of promoters comprises bidirectional promoters. These regulatory regions fall between two genes and regulate transcription of the genes in opposite directions from the promoter region, i.e. the bidirectional nature contrasts that of a typical uni-directional promoter. These promoters represent a subclass of the larger gene of promoter sequences [2,3]. Previous studies have shown that bidirectional promoters are enriched in the genome [4] (Adachi and Lieber 2003), tend to be co-expressed [5] and bind *ets *proteins [2,6,7]. One approach to elucidating the molecular mechanisms regulating bidirectional promoters is to map the content of CPEs. Since co-expression of both genes happens more frequently than random events [5], an explanatory model would suggest symmetry of the promoter elements near the TSSs. This manuscript addresses the distribution of CPEs in the human bidirectional promoters using computational analyses of current large-scale experimental datasets as well as motif analyses. We address the issues of C+G content, patterns of transcription initiation and symmetry of CPEs near the TSSs.

## Results

### Core promoter elements

#### TATA box

The earliest descriptions of functional promoter elements focused on the importance of a TATA-motif to recruit the essential RNA polymerase II molecule to the transcription start site (TSS). We now understand that the TATA-centric view of promoters represents only a minor proportion of promoters in eukaryotic cells [8].

**Figure 1:**
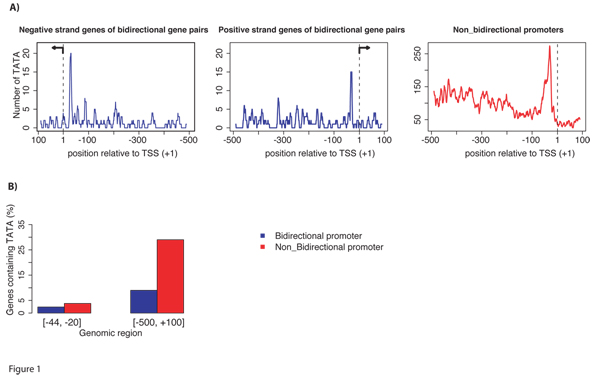
**TATA motifs in bidirectional promoters**. (A) TATA motifs were mapped in the 500 bp regions upstream of TSSs and 100 bp downstream for bidirectional promoters and nonbidirectional promoters. (B) The percentage of genes with TATA motifs as measured at the functional position at -30 bp upstream of the TSS. A range from -44 to -20 was used to accommodate some error in the annotations. The full promoter regions from positions -500 to +100 contains many occurrences of TATA motifs, however based on the characterized mechanism of the TATA motif, these are false positive predictions.

By scanning non-bidirectional and bidirectional promoters (see the definitions in the Methods section), we found that 29% of non-bidirectional promoters and 9% of bidirectional promoters contained a TATA motif. This result was consistent with previous reports [5], which suggested that the TATA occurrence was depleted in bidirectional promoters compared to the genome average. The prevalence of TATA motifs in non-bidirectional promoters was statistically significant over that expected by chance; however, in bidirectional promoters did not vary significantly from the rate expected by chance. Reducing the range of the searchable window to a region  surrounding the -30 position, which is essential for proper TATA-box function (see the Methods section), we found that the occurrence of TATA boxes decreased to 3.8% for non-bidirectional promoters and 1.2% for bidirectional promoters, respectively (Fig. 1). The TATA motifs peaked at the functional location (-30 position) in both the non-bidirectional and bidirectional promoters. By this approach, the presence of the TATA motif in both types of promoters was significantly larger than expected by random chance, which occurred at 0.33% and 0.08% (p-value < 0.0002), respectively. Thus searching only the known functional position of the motif filtered out a majority of false positive predictions. Although the TATA motif in bidirectional promoters was lower than the genome-average, it was clearly present in a select group of bidirectional promoters. Of these, the data showed a strong enrichment for histone genes. A p-value for enrichment was 8.38e-09 compared to a random set of sequences.

#### Downstream promoter element (DPE)

**Figure 2:**
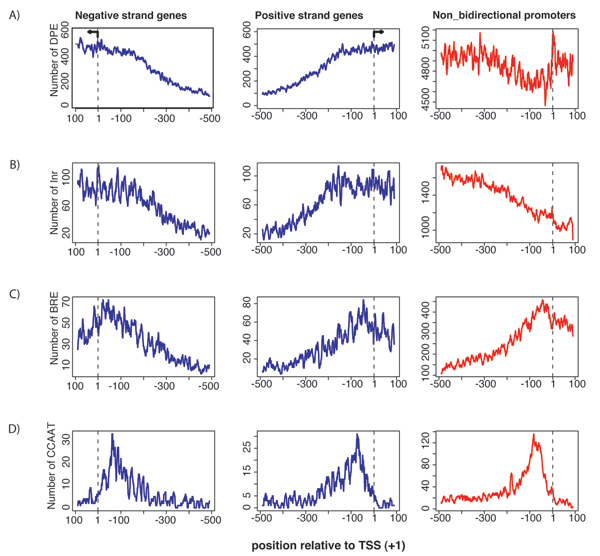
**CPEs in bidirectional promoters**. Core promoter elements include TATA, INR, BRE, and CCAAT motifs (A-D). Elements were mapped in the full promoter region. A dashed line at position +1 indicates the TSS. Bidirectional promoters are plotted in blue for negative and positive strand genes. Nonbidirectional promoters are plotted in red.

DPE is a downstream promoter element that is conserved from *Drosophila *to human [9]. The DPE motif is usually located at the downstream position +30 relative to the transcription start site. We found that 46.6% of bidirectional promoters and 50.6% of non-bidirectional promoters contained this motif at the functional position (Fig. 2A). The presence of DPE in both type of promoters was significantly larger than expected by random chance, which was 15% and 16% (p-values < 2.2e-16), respectively.

#### INR element

The INR [10] is a conserved sequence that encompasses the TSS, which functions to direct accurate transcription initiation either by itself or in conjunction with TATA or DPE. We found that 25.3% of bidirectional promoters contain the INR motif while 30.8% of non-bidirectional promoters contained this motif at the functional position (Fig. 2B). The presence of the INR in both type of promoters was significantly larger than the frequency expected by random chance, which was 9.28%% and 14.10%% (p-values < 2.2e-16), respectively.

#### TFllB recognition element (BRE)

The BRE is located immediately upstream of TATA box [11] of some promoters containing TATA. We found that 16.5% of bidirectional and 11.1% of non-bidirectional promoters contained this motif at the functional position (Fig. 2C). The presence of BRE in both types of promoters was significantly larger than the frequency expected by random chance, which was 5.2% and 2.1% (p-values < 2.2e-16), respectively.

#### CCAAT

The CCAAT motif represents a consensus sequence that occurs upstream of the TSS by 75–80 bases. We found that 12.9% of bidirectional promoters contain CCAAT element while 6.9% of non-bidirectional promoters contained this motif at the functional position (Fig 2D). Presence of CCAAT in both types of promoters was significantly larger than frequency expected by random chance 0.66% and 0.91% (p-value < 2.2e-16).

#### C+G content and CpG-islands

**Figure 3:**
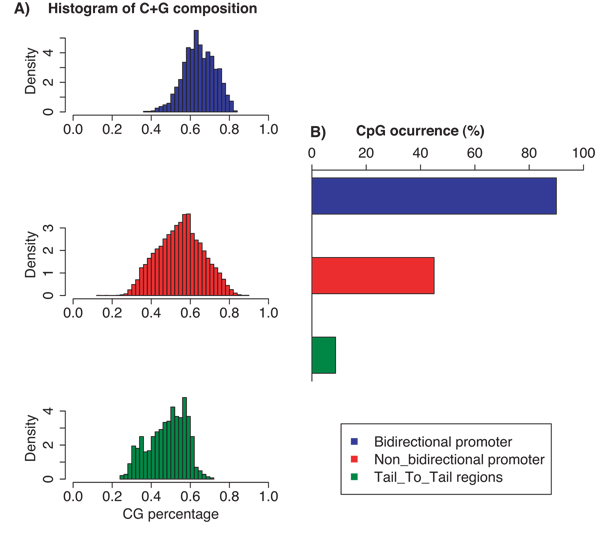
**CG nucleotide bias in bidirectional promoters**. **(A) **C+G content plotted as a histogram of the dinucleotide density. Plots are stacked with bidirectional promoters on top and Tail_to_tail regions on the bottom. (B) The CpG-island content of these same categories of promoters.

Promoters have high C+G contents compared to the other noncoding regions such as the intergenic regions between the 3' ends of genes i.e., tail_to_tail regions (see Methods). The average percentage of C+G nucleotides in bidirectional promoters, non-bidirectional promoters and tail-to-tail regions was 64%, 55% and 45%, respectively. The C+G percentage of each category (Fig. 3A) showed that 70.8% of bidirectional promoters had C+G content exceeding 60%, while only 8.3% of tail-to-tail regions had C+G content exceeding 60%. Consistent with a high C+G content, bidirectional promoters had a significant enrichment of CpG-islands (Fig. 3B). CpG-islands were present in 90% of bidirectional promoters compared to 45% of non-bidirectional promoters and only 9% of tail-to-tail regions.

**Figure 4:**
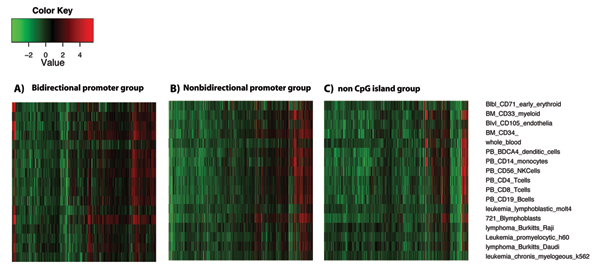
**Expression profiles of CpG-island promoters**. Expression profiles for 17 blood-related samples were analyzed. The data are divided into (A) bidirectional promoters with CpG-islands (B) nonbidirectional promoters with CpG-islands (C) nonbidirectional promoters without CpG-islands. Individual columns in the plot represent genes, whereas cells and tissues are on the vertical axis. Expression is relative to the median value from >16,000 genes available in the human GNF dataset. Red indicates expression above the median, green is below, black is equivalent.

The correspondence between CpG-islands and gene expression was measured for bidirectional promoters and non-bidirectional promoters (Fig. 4A). In 17 human tissues of blood-cell identity, 31% of genes with bidirectional, CpG-island promoters had higher expression than the median data from 16,000 genes in 79 tissues. A slightly lower percentage of 24% was recorded for genes with non-bidirectional, CpG-island promoters (Fig. 4B). In contrast, only 14% of genes lacking CpG-islands showed expression above the median value (measured on non-bidirectional promoters only) (Fig. 4C). The percentage of genes with lower than median expression values were 11%, 18% and 24% for bidirectional promoters and non-bidirectional promoters containing CpG-islands and non-bidirectional promoters lacking CpG-islands, respectively. Thus the presence of a CpG-island corresponded to a trend toward higher gene expression from bidirectional promoters.

### ChIP-seq data

Increasingly, high-throughput experimental studies are providing a wealth of information that is useful for deducing biologically relevant themes. Assays such as ChIP-chip or ChIP-seq are powerful investigative tools for revealing the presence of a protein bound to DNA. The cost and labor involved with such studies are large; however the significance of these experimental results far exceeds any other method for obtaining binding information at this scale. For example, ChIP-chip data revealed the binding of RNA polymerase II at the collection of active promoters in the cell, providing a snapshot of the inner workings of the cell [12]. We used the ChIP-seq data of Barski et al. [13] for RNA polymerase II to determine which promoters were occupied by the transcription machinery.

**Figure 5:**
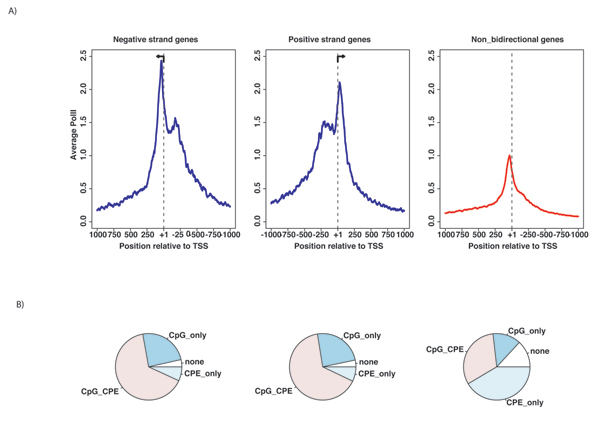
**RNA POLII sequence tags at bidirectional promoters**. (A) Data are separated into negative and positive strand genes. Bidirectional promoters are plotted in blue, whereas nonbidirectional promoters are in red. RNA POLII tags are averaged by the total number of promoters. (B) Pie charts of the promoter elements in each dataset. CpG-island = CpG, Any core promoter element = CPE.

These data showed that the occupancy of RNA polymerase II was over 2-fold greater at bidirectional promoters than at non-bidirectional promoters. This result is consistent with the idea of two active transcription forks in the bidirectional promoter region. Moreover, the data suggested that these regions recruited RNA Pol II efficiently. The higher proportion of expression values in CpG-island bidirectional promoters compared to nonCpG-island promoters (Fig. 4) was consistent with the higher recruitment of RNA POLII shown here by the ChIP-seq data (Fig. 5A). A slightly higher number of PoIll tags appeared at the negative strand genes than the positive strand genes in the bidirectional gene pairs. This observation prompted the analysis of Core Promoter Elements (CPEs) in both negative strand genes and positive strand genes (Table 1). Most elements were simultaneously represented at the left and right TSSs. Notably, nonbidirectional promoters contained a larger than expected proportion of promoters with no core promoter elements (Fig. 5B).

**Table 1:** Core promoter elements at left and right TSSs

	CpG	TATA	DPE	INR	CCAAT	BRE
Overlap TSS of left gene	113	17	344	244	78	167
Overlap TSS of right gene	103	14	357	245	83	161
Present at both TSS	1009	1	301	109	100	67

### Asymmetry of TATA occurrence in bidirectional promoters

**Figure 6:**
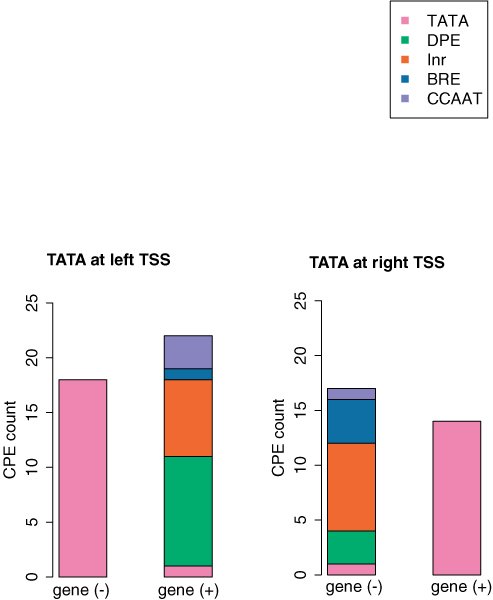
**Assymmetry of TATA motifs in bidirectional promoters**. TATA motifs present at the left and right TSS are plotted separately. When a TATA-motif was detected, the functional TATA position was assessed at the other TSS. Only one gene pair showed a TATA at both positions. Other CPE motifs were mapped to explain regulatory control in the absence of the TATA motif.

We mapped all bidirectional promoter regions for the presence of TATA motifs with respect to the left and right TSSs. Only one regulatory region had a TATA motif on the left and right sides at the correct positions (i.e -30). This result indicated an asymmetry of regulatory elements utilizing TATA motifs in bidirectional promoters (Table 1). Furthermore, we looked the occurrence of other CPEs at the TATA-depleted TSS (Fig. 6). All forms of CPE elements were found at the TATA depleted TSSs; showing no balanced counterpart to the TATA motif.

### CAGE

Recently sets of transcripts with precise initiation sites have been produced and mapped onto their positions in the genome. This experimental technique, known as cap-trapping or CAGE[14], precisely defines TSSs by capturing all transcripts at their first nucleotide (recognized by its methylated *cap*). This cap is "worn" at the beginning of the transcript, which corresponds to the "head" or beginning of the gene. Data generated by cap-trapping assays promise to significantly advance our knowledge of the transcriptome in any given cell type, refine our knowledge of the start sites of genes, and, by inference, pave the way for promoter analyses  that examine the sequences immediately upstream and downstream of the captured TSSs.

**Figure 7:**
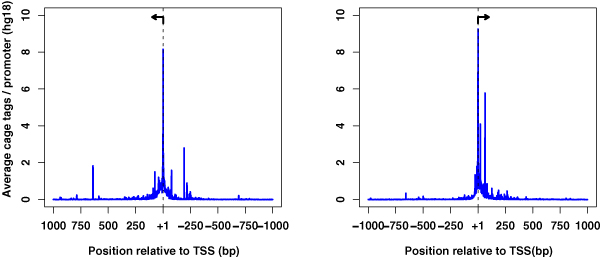
**CAGE tags at bidirectional promoters**. CAGE tags were mapped according to their sequence identity on the negative or positive strands. Within a 20 bp regions surrounding the TSS, one dominant peak is detected for each strand. CAGE tags represent an average of the number of promoters in the analysis.

The bidirectional promoter dataset was validated by CAGE experimental evidence. The main peak of CAGE tags occurred at the mapped TSSs for bidirectional promoters of negative and positive strand genes (Fig 7). Additional short peaks in the CAGE  data indicated that a few minor initiation sites were present. In contrast to the characterized, broad patterns of initiation at CpG-island promoters [1], bidirectional promoters have very distinct sites of initiation. Mapping the number of paired (left and right) transcripts, we found evidence for 615 co-expressed genes out of 1,366 gene pairs.

## Conclusion

Bidirectional promoters comprise a diverse set of core-promoter regulatory elements. A subset of these promoters contain TATA motifs, with notable enrichment at histone promoters. We did not find a balanced representation of TATA at both the left and right TSSs of gene pairs, including the histone genes. This result indicated that bidirectional promoters can employ different methods of regulation within a pair of genes. Furthermore, we found that 45% of genes were co-expressed – by virtue of the CAGE tags. This approach excluded signals from downstream alternative promoters, which complicate measurements in microarray analyses; confirm that a large proportion of these promoters are co-expressed from the neighbouring TSSs. Bidirectional promoters coincided with CpG-islands more often than non-bidirectional promoters. This genomic feature may play a significant role in marking these regions as promoters as well as participating in Pol II recruitment.

## Methods

### Genomic regions

We downloaded 56,722 protein-coding gene annotations from UCSC genome browser hg18 database. These collapsed into 25,147 unique and non-overlapping gene clusters. Of these, 1,369 bidirectional gene pairs were present, defining bidirectional promoters (for 2,738 genes). Each gene in a bidirectional gene pair formed a head-to-head arrangement with its closest neighbour and the intergenic distances between the TSS of a gene and its neighbour had to be within 1,000 bp [2]. After excluding those pairs with too large an intergenic distance and those with anti-sense overlap at the 5' ends of the transcripts, we obtained 13,302 genes, which did not form head-to-head arrangement with the closest neighbour. These were designated non-bidirectional promoters. We also defined a negative control set. When a gene and its closest neighbour were transcribed in convergent directions, ending within 1000 bp of each other, they were designated as tail-to-tail regions.

### Core promoter elements analysis

For bidirectional promoters we extracted the intervening DNA sequence between the TSSs and extending 100bp downstreanm of the TSS for each gene. For non-bidirectional promoters, sequence was extracted 500bp upstream and downstream 100bp of  the TSS site. For sequences between the 3' ends of tail-to-tail gene we extracted the region between the genes plus 100bp into the genes. We mapped the distributions and frequencies of  five regulatory sites: TATA, CCAAT, DPE, INR and BRE in these three type of genomic regions. Furthermore, we measured the occurrence of these promoter elements within restricted intervals that are known to be functional leaving a small window on either side for slightly imprecise localization. We searched TATA [A|T]A [A|G|T] for TATA at the regions between -40 to -20, [A|G] [A|G]CCAAT [A|C|G] [A|G] for CCAAT between -108 and +9, [A|G|T] [C|G] [A|T] [C|T] [A|C|G] [C|T] for DPE between +24 to +34 for DPE, [C|T] [C|T]AN [A|T] [C|T] [C|T] for INR -15 to +15, and [G|C] [G|C] [G|A]CGCC for BRE between -49 to -18. Then the observed occurrence rate was calculated for each promoter element respectively. Using the nucleotide frequency in the promoter sequences, we obtained probability of finding a CPE by chance per promoter. The χ^2 ^4test was performed to determine whether the difference between the occurrence rate by random events and by measured observation was significant or not.

### Microarray expression data

Gene Expression Altas2 data is from the USCS Human Genome Browser. The dataset consists of expression data for 79 human tissues produced by Genomics Institute of the Novartis Research Foundation (GNF) [15]. Compared to the median expression ratio, values larger and smaller than 1 were classified as over-expression under-expression, respectively.

### ChIP-seq data

Tag density for RNA polymerase II binding sites were obtained by the total number of Pol II tags divided by number of promoters.

### Cap analysis of gene expression (CAGE)

CAGE tags are available at the Riken website . The dataset contains CAGE tags in 1,057,486 positions of hg17 assembly. After converting the genomic coordinates of bidirectional promoters in hg18 to hg17 assembly by liftover, we mapped the CAGE data to the bidirectional promoters.

## Competing interests

The authors declare that they have no competing interests.

## Authors' contributions

Mary Q. Yang and Laura Elnitski conceived of the analysis and Mary Qu Yang performed the experiments. Both authors contributed to the writing of the manuscript.
